# Safety Assurance of a High Voltage Controller for an Industrial Robotic System

**DOI:** 10.1007/978-3-030-63882-5_4

**Published:** 2020-10-27

**Authors:** Yvonne Murray, David A. Anisi, Martin Sirevåg, Pedro Ribeiro, Rabah Saleh Hagag

**Affiliations:** 8 Federal University of Pernambuco, Recife, Brazil; 9 Western Norway University of Applied Sciences, Bergen, Norway; 10 Department of Mechatronics, Faculty of Engineering and Science, University of Agder (UiA), Grimstad, Norway; 11 Robotics Group, Faculty of Science and Technology, Norwegian University of Life Sciences (NMBU), Ås, Norway; 12 Department of Computer Science, University of York, York, UK

**Keywords:** Formal verification, Model checking, High Voltage Controller (HVC), Industrial robots

## Abstract

Due to the risk of discharge sparks and ignition, there are strict rules concerning the safety of high voltage electrostatic systems used in industrial painting robots. In order to assure that the system fulfils its safety requirements, formal verification is an important tool to supplement traditional testing and quality assurance procedures. The work in this paper presents formal verification of the most important safety functions of a high voltage controller. The controller has been modelled as a finite state machine, which was formally verified using two different model checking software tools; Simulink Design Verifier and RoboTool. Five safety critical properties were specified and formally verified using the two tools. Simulink was chosen as a low-threshold entry point since MathWorks products are well known to most practitioners. RoboTool serves as a software tool targeted towards model checking, thus providing more advanced options for the more experienced user. The comparative study and results show that all properties were successfully verified. The verification times in both tools were in the order of a few minutes, which was within the acceptable time limit for this particular application.

## Introduction

Formal verification provides an extra level of assurance by verifying the logic of a system and making sure that it works in accordance to its specifications in every situation. This will ideally help identify potential pitfalls in a much earlier phase of the development cycle [1]. The two main approaches are model checking [2] and theorem proving [3]. Application of formal methods in industrial use cases is an important supplement to the traditional testing and safety risk identification and mitigation actions which are already taking place [4]. Obtaining sufficiently high testing coverage in complex industrial systems may be very time consuming and tedious. In practice, it may even be impossible to test for every scenario or situation, which means that testing could possibly fail to reveal potential safety critical bugs and errors. As a testimony of this, Sect. 2 outlines some previous errors that went by undetected by traditional testing methods.

**Fig. 1.**
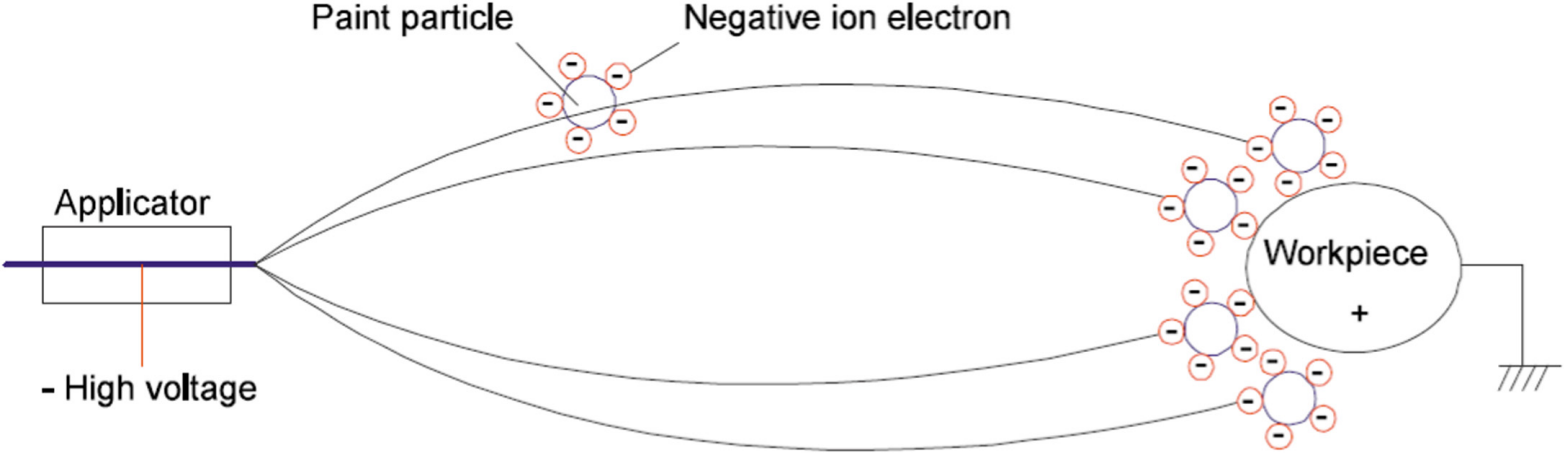
In electrostatic painting, high voltage (approximately 50–100 kV) charges the paint particles at the applicator. The particles follow the lines of the electrostatic field from the applicator (cathode) to the earthed object (anode).

Industrial paint robots use High Voltage (HV) to perform electrostatic painting, where particles are electrically charged and attracted to the grounded paint object, as seen in Fig. 1. In this way, painting quality is ensured while paint consumption and costs are minimized. However, HV also poses certain risks, particularly in explosive atmospheres where potential discharge sparks may cause ignition. Fire in the painting cell will result in costly production delays, as well as damage to the equipment. Therefore, it is of great importance that the High Voltage Controller (HVC) is working as intended, and there are strict rules to ensure the safety of the system and personnel. These include both software-based safety layers such as over current- and max current detection, as well as physical safety layers based on (optical) fencing, minimum clearance distances, and use of safety clothes such as anti-static shoes and gloves.

An HVC used in an industrial paint robot has been provided for this case study. After passing the traditional quality assurance and testing procedures, some undesired system behavior was discovered. Thus, even though risk mitigation plans were identified systematically (e.g., using HAZID/HAZOP), and thorough testing on both component and system level had been conducted, some errors still managed to go undetected. The undetected errors had in common that they happened when certain conditions and situations happened in a very specific order, and that specific series of events had not been tested. This gave a strong motivation for performing a formal verification of the HVC, in order to ensure both that the found errors had been fixed and that there were no other situations where the same errors could occur.

The logic of the C++ code of the HVC can be modelled as a finite state machine, which means that model checking is an appropriate method for the formal verification of the properties. The methodology of model checking has some apparent advantages that fits industrial applications very well; it is a rather general verification approach which has some commercial-grade, high-performance model checkers available. It provides diagnostic information (counter-example) that can be used for debugging purposes, is easier to integrate with existing development and engineering practices and last but not least; is more intuitive and familiar to most practitioners than theorem proving [2].

In this work, two different software tools have been used to model and verify the HVC system. The first is Simulink Design Verifier (SDV) by MathWorks [5] and the second is RoboTool [6, 7], developed by the RoboStar group at University of York. Here-within, Simulink was chosen as a low-threshold entry point since MathWorks products are well known to most practitioners. RoboTool serves as a software tool targeted towards model checking, thus providing more advanced options for the more experienced user. In addition to presenting and analyzing an interesting industrial use case considering formal verification of the safety aspects of the HVC unit of a paint robot, the main objective of this work is to do a comparative study of the software tools with regards to functionality, usability and effectiveness, e.g., modelling, validation and analysis time.

Application of formal verification methodology within the control and robotics community have mainly adopted the hybrid system and automata framework of Alur *et al.* [8, 9]. In this setting, finite- and infinite-time reachability constitute the main verification tools, but unfortunately turn out to be an undecidable problem in general, leaving conservative set approximation as the only viable approach [10, 11]. Hybrid automata theory also assumes having infinite accuracy and instantaneous reaction which serves as a noticeable discrepancy to the real system and implementation; potentially invalidating the formal verification results [12]. Narrowing down to industrial paint robots, [13] considers formal verification of the paint spraying use case using ARIADNE tool for reachability analysis. The focus here is solely on parametric design verification. To the best of our knowledge, there is no prior art considering formal verification of the safety aspects of the HVC unit of an industrial paint robot, which is the focus of the paper at hand.

The remaining of this paper is structured as follows. Section 2 details the HVC system and previous errors that were not found by traditional testing methods. It also contains formulation of the properties to be formally verified. Section 3 presents a simplified finite state machine of the HVC. Section 4 explains how the state machine was modelled in RoboTool and SDV, and how these tools were used to verify the properties. Finally, Sect. 5 provides some discussion and conclusions, as well as a comparison between the two tools. Additionally, suggestions for further research is presented.

## HVC and Previously Detected Errors

**Fig. 2.**
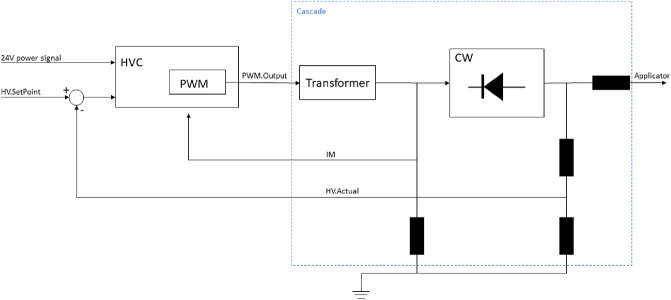
Block diagram of one part of the paint robot, containing the HVC.

A simplified block diagram of the part of the paint robot that contains the HVC can be seen in Fig. 2. Here, the $r(t)= HV\_SetPoint$ signal is used as reference for the desired voltage level on the HVC, while the 24 V power signal provides the HVC with electrical power. The HVC module runs the control loop and associated control logic. The $u(t) = PWM\_Output$ signal shows the calculated value for the high voltage regulator, from 0 to 100%, which is then increased in the transformer. In the Cockcroft–Walton (CW) generator, there are several voltage doubling circuits, and the voltage is rectified and further increased, before arriving to the applicator. Here, $y(t) = [IM,\quad HV\_Actual ]^T$ denote current and voltage measurements, respectively, which are fed back into the HVC.

**Fig. 3.**
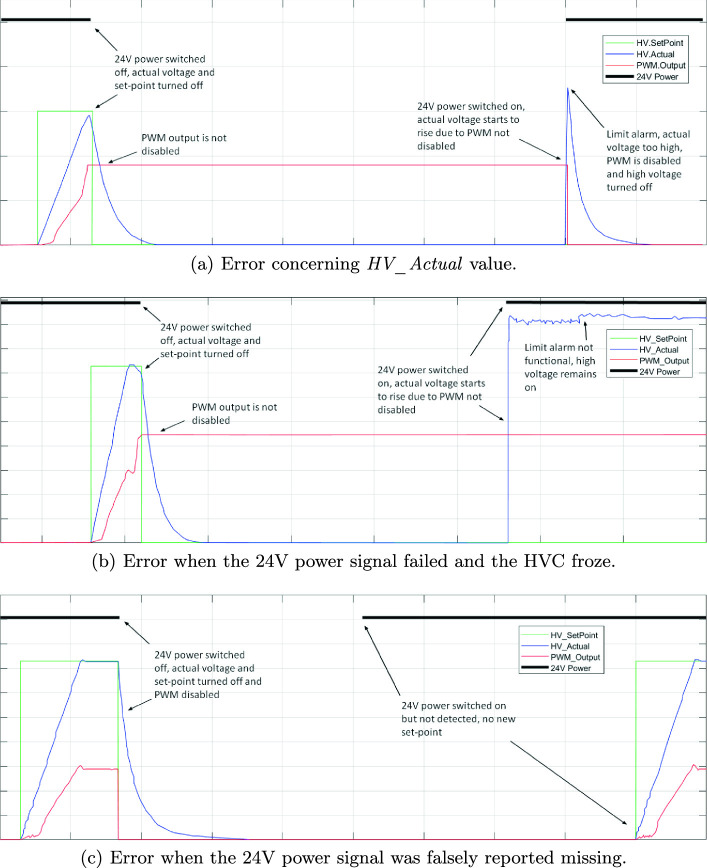
Some errors that were not discovered through traditional testing of a previous version of the HVC software. These shed light on the need for the industry to adopt formal verification methodology when developing safety critical systems. The x-axis unit is time, while the y-axis units are kV for the high-voltage signals, percent for the PWM and binary on/off for the 24 V power signal. Here, the schematic representation and inter-relation between the signals are in focus, not the exact values.

Referring to Fig. 3, the previous version of the HVC had two main issues, containing several variations:

1. Issues with the actual voltage level on the HVC, *HV_Actual*:
  - Both the set-point and *HV_Actual* had a non-zero value, but they differed from each other. The HVC board did not respond to any further set-point changes, and had a constant actual value.
  - There was no set-point, but *HV_Actual* had a non-zero value.
  - There was a set-point, but *HV_Actual* was still zero.
2. Issues with the 24 V power signal:
  - The HVC sometimes reported the 24 V power signal missing, even though it was actually present, resulting in a deadlock.
  - Sometimes, an additional bug also occurred, where the HVC froze when the 24 V power signal failed. In that case, the HVC limit supervision did not disable PWM, and the HVC continued to set out high voltage until it was reset or powered off. This can be seen graphically in Fig. 3(b).

The issues 1a–c, regarding the *HV_Actual* value, occurred if the 24 V power signal was switched off when the set-point had a value other than 0kV. When this happened, the set-point was turned off due to the missing 24 V power signal, and the *HV_Actual* signal dropped to 0kV. However, the problem was that the Pulse Width Modulation (PWM) output which drives the cascade, *PWM_Output*, was not disabled. Thus, when the 24 V power signal was switched on again, the PWM fed the cascade, and the voltage output from the cascade increased. The HVC limit supervision caught this voltage rise, and disabled the PWM, since the resulting voltage was too high. This problem can be seen graphically in Fig. 3(a).

As for issue 2a, the 24 V power signal missing issue was due to a deadlock in the HVC that resulted in the controller reporting the 24 V power signal missing, even though it was actually present. This can be seen graphically in Fig. 3(c).

Upon rectifying these observed issues in a later software upgrade, the task at hand is to run formal verification on the upgraded software in order to ensure both that the previously found errors have been fixed and that there are no other situations where similar errors could occur.

### Properties for Formal Verification

In this section, the set of properties that are to be formally verified will be presented and discussed. Most of them are rather natural and generic properties to be fulfilled by any feedback controller tracking a set-point reference. Also, the previously detected errors provide a testimony of which properties that are necessary to formally verify in order to ensure that they will not happen again, under any circumstances.

As one of the most profound properties of any feedback controller, it is reasonable to require that *HV_Actual* should always follow *HV_SetPoint*. This deserves particular attention in cases with residual voltage as depicted in for instance Fig. 3(b). To formalize this, since all voltages here are non-negative, the following implications were considered:

1 $$\begin{aligned} \begin{aligned} HV\_SetPoint = 0&\rightarrow HV\_Actual = 0 \\ HV\_SetPoint> 0&\rightarrow HV\_Actual > 0 \end{aligned} \end{aligned}$$

Notice that by logical transposition of (1), the following implications also hold true:

$$\begin{aligned} \begin{aligned} HV\_Actual> 0&\rightarrow HV\_SetPoint > 0 \\ HV\_Actual = 0&\rightarrow HV\_SetPoint = 0 \end{aligned} \end{aligned}$$

As a result, the first property boils down to verification of:

$$\begin{aligned} HV\_SetPoint = 0 \longleftrightarrow HV\_Actual = 0. \end{aligned}$$

To avoid residual effects and windup type of behavior in the HVC, it is also reasonable to verify that both *PWM_Output* and *HV_SetPoint* are set to 0 whenever the 24 V power signal, and thereby the HVC-module, is switched off.

Additionally, in order to increase the confidence in the correctness of the model, it is customary to verify that the HVC state machine is not able to go into deadlock, and that all the states in the finite state machine are reachable, *i.e.*, the state machine has no dead logic.

To sum up, the properties chosen for the formal verification are:

1. That the actual system voltage always follows the set-point:
  $$\begin{aligned} HV\_SetPoint = 0 \longleftrightarrow HV\_Actual = 0 \end{aligned}$$
2. That *PWM_Output* is set to 0 whenever the 24 V power signal is off:
  $$\begin{aligned} 24\,\mathrm{V}\_Power = 0 \rightarrow PWM\_Output = 0 \end{aligned}$$
3. That *HV_SetPoint* is set to 0 when the 24 V power signal is switched off:
  $$\begin{aligned} \mathrm{24}\,\mathrm{V}\_Power = 0 \rightarrow HV\_SetPoint = 0 \end{aligned}$$
4. That the state machine is not able to go into deadlock
5. That all states in the state machine are reachable.

## Finite State Machine Modelling

**Fig. 4.**
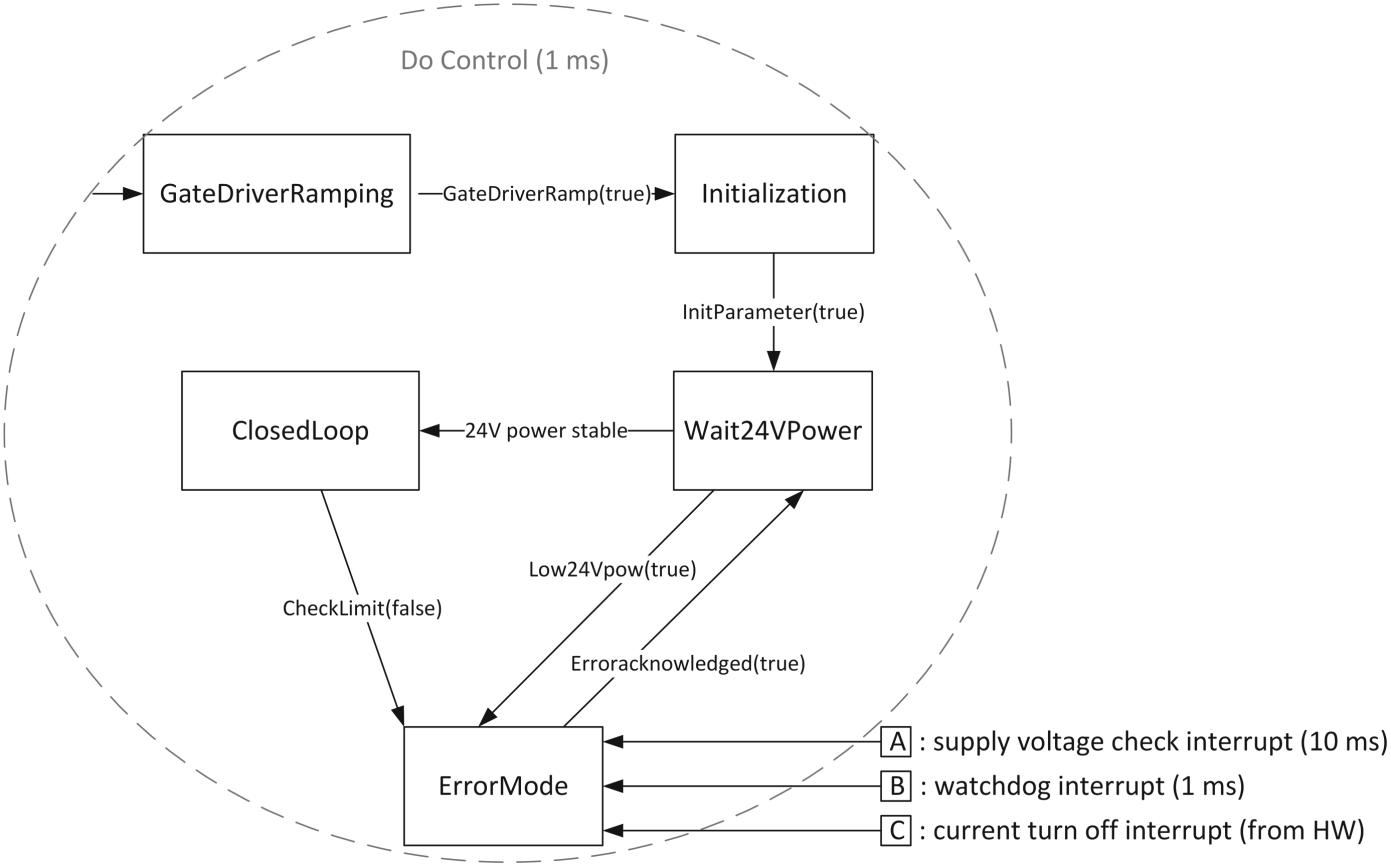
Finite state diagram of the High Voltage Controller (HVC).

In order to perform model checking on the HVC, its functionalities were modeled as a finite state machine. This section presents the general finite state machine, which was directly derived from the implemented C++ code and depicted in Fig. 4. This state machine was then modelled and verified in RoboTool and Simulink. This is the topic of Sect. 4.

In the state  , which is the state the HVC first enters when it is switched on, the PWM duty-cycle is ramped up gradually to ensure stability and gradual increasing of current and voltage. Then, in the  state, initial parameters are set, as well as upper and lower limits for the high voltage.

After the  and  steps are successfully finished, the state machine enters the  state. When the HVC has 24 V power switched on and stable, the system enters the  state. This is the ideal state for operation, and is where the controller is regulating the voltage in relation to the set-point. In case the voltage is breaching the upper or lower limits, the state machine moves from  to  .

There is also a possibility to go straight to  from any of the other states, if certain variables are set or any watchdogs or interrupts are triggered. For instance, an interrupt is triggered if the supply voltage is below a certain threshold, and another is triggered if *HV_Actual* is above or below the upper and lower limits, respectively. Getting out of  requires manual acknowledgement of the occurred errors.

## Model Checking

The finite state machine created from the C++ code of the HVC has to be modelled in a model checking tool in order to verify the selected properties. To this end, two different tools are adopted, in order to compare and evaluate their functionality and effectiveness. In this work, the finite state machine has been modelled in RoboTool [6] using the modelling language RoboChart, and in SDV [5] using the modelling language Stateflow. This is done in Sect. 4.1 and 4.2 respectively.

### Model Checking in RoboTool

RoboTool and its modelling language RoboChart are specifically designed to model robotic systems for formal verification [6]. The tool automatically generates Communicating Sequential Processes (CSP) [14] proof models, which are verified using the Failures-Divergences Refinement (FDR) model-checker [15, 16].

When using RoboChart for modelling, it is important to be aware that the model has to be of a higher abstraction level than models used for dynamic simulation. Capturing the behaviour from the C++ code in an abstract modelling paradigm like RoboChart can be challenging, especially for practitioners who are used to work with input-driven, dynamic simulations. Instead, the model is analyzed by the verification tool by only assuming bounded data-types and going through all possible transitions in order to verify or disprove a property. Specific values for variables or inputs could be used, but this would restrict the range of values provided by the bounded data type. Thus, keeping a high level of abstraction during the modelling process is essential for getting a meaningful result from the model checking.

**Simplifications to Reduce Verification Time.** State-space explosion is a well-known issue for model checkers. For this reason, some lower level functionalities from the C++ code were simplified. As an example, the ramping function in  was modelled simply by staying in the state for a certain number of time steps, representing the ramping time. This simplification, justified by the fact that  occurs before the initialization state and therefore does not influence neither the verification properties nor results, greatly reduced the verification time.

**Fig. 5.**
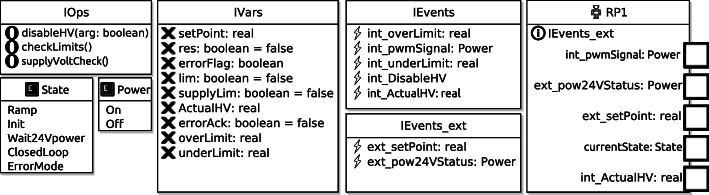
Components and enumerated types used in the RoboChart model.

**The Model.** For modelling the state machine as closely as possible to Fig. 4, the software operations (IOps), variables (IVars), events (IEvents), external events (IEvents_ext) and a robotic platform (RP1), were specified as shown in Fig. 5. The robotic platform (RP1) is an abstraction of the physical system, and only uses events that require communication with the system (IEvents_ext), whereas the other events and variables are internal to the software. For more details about the language structure and semantics used in RoboTool, please consult [6, 17].

**Fig. 6.**
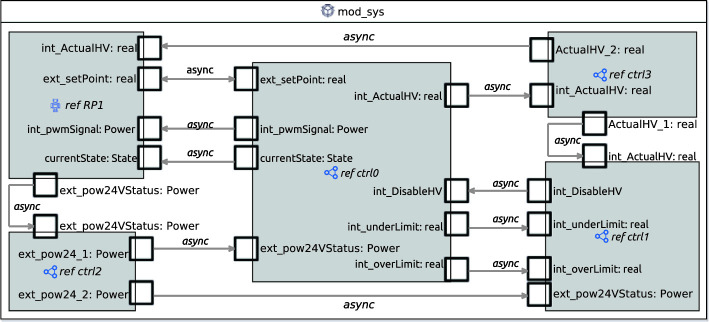
RoboChart module mod_sys defining the connections between controllers and the robotic platform. Controller ctrl0 contains the main State_machine, a recast in RoboChart of the state machine presented in Fig. 4. The watchdogs have been combined into one state machine, defined inside controller ctrl1. Controllers ctrl2 and ctrl3 are used purely for relaying events int_ActualHV and ext_pow24VStatus to other controllers.

Figure 6 shows the RoboChart module mod_sys, which defines the connections between the controllers (ctrl0-3), and the robotic platform (RP1). In RoboChart, connections with the platform are asynchronous, indicated by the keyword async, as interactions with the hardware cannot be refused, only ignored [7, p. 3110].

**Fig. 7.**
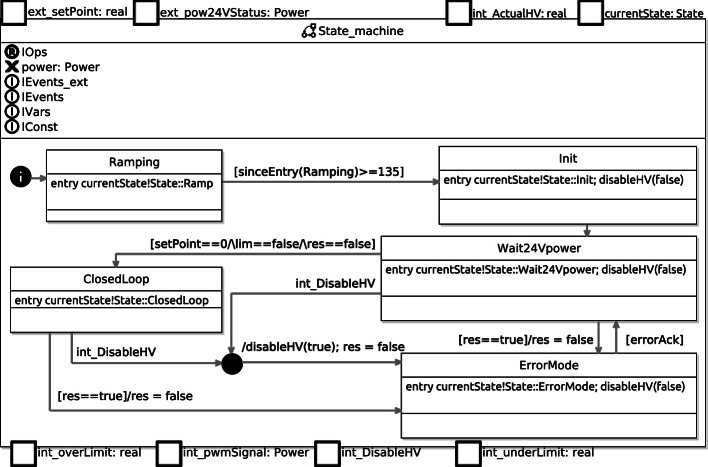
Main State_machine corresponding to that of Fig. 4 recast in RoboChart, with the internal behaviour of composite states (other than Ramping) elided.

Using the components from Fig. 5, a state machine model was created using the graphical user interface in RoboTool. An overview of the states and the transitions between them can be seen in Fig. 7.

**Fig. 8.**
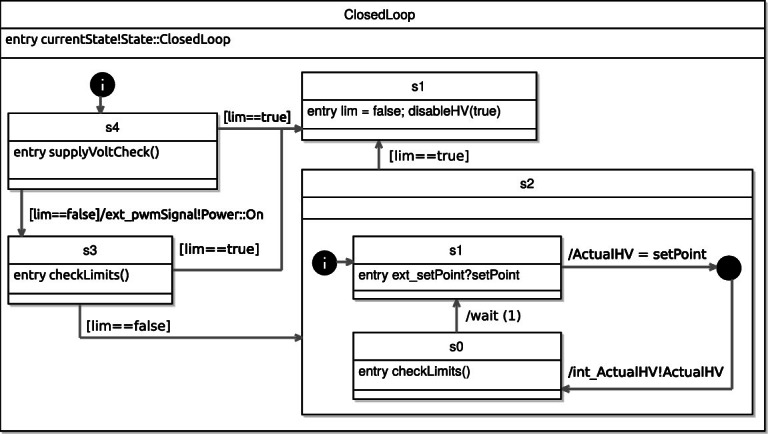
ClosedLoop state of State_machine.

**Fig. 9.**
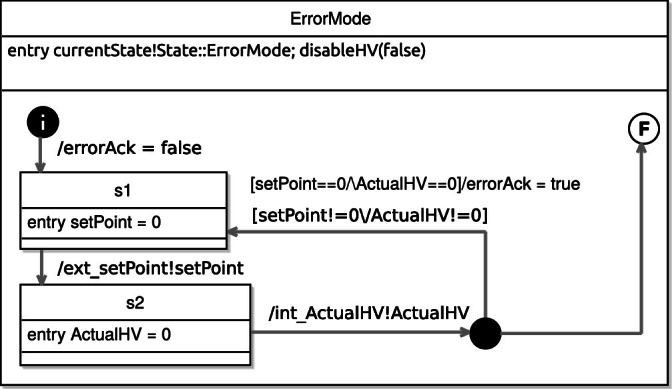
ErrorMode state of State_machine.

All of the top-level states have an entry action, which indicates the current state via the typed event currentState. This is useful for analysis of properties that are only applicable in certain states. The detailed view of the two most important states (ClosedLoop and ErrorMode) can be seen in Fig. 8 and 9.

**Verification of Selected Properties.** In order to verify the properties from Sect. 2 in FDR, the properties had to be formally written in CSP. To specify simple assertions, such as deadlock freedom, RoboTool provides a simple textual domain-specific language. More complex properties, however, have to be specified directly in  , the machine-readable version of CSP. A full account of the CSP specifications for all properties discussed in what follows can be found online^1^.

The first property to verify, P1 as described in Sect. 2, is that *HV_Actual* follows *HV_SetPoint*. Due to the necessary simplifications that were made when modelling *HV_Actual* and *HV_SetPoint*, a binary version of property P1 had to be considered, where the signals *HV_Actual* and *HV_SetPoint* are considered to be either on or off. Notice that this modification is without loss of generality.

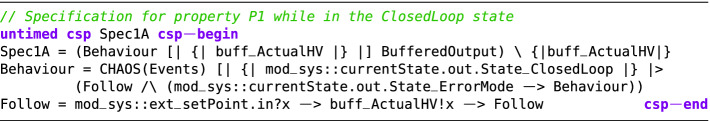



The CSP process  is a parallel composition  of  , synchronising on the event  , with  (omitted) that accounts for the asynchronous communication of the output int_ActualHV in mod_sys (as shown in Fig. 6), and where the event  , used to communicate with the buffer, is hidden  .  behaves as  , that can perform any event in the set  (of all events) non-deterministically until  the event  happens, and then behaves as  . Events in the CSP semantics of RoboChart are named according to the hierarchy, where  is a delimiter, and have a parameter  or  to indicate whether an event is an input or output. This states that when the system is in the ClosedLoop state, then it behaves as  , which establishes that the input  value  received via ext_setPoint is followed by an output  of the same value via  , and a recursion.  may be interrupted  if an error occurs, indicated by the currentState event with value ErrorMode. To ensure appropriate behaviour in that state, we specify the following property.

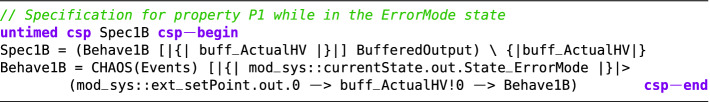



Similarly to  , in  we have that in the ErrorMode state the value of the *HV_SetPoint* is 0, in this case observed as an output of the system via ext_setPoint, and that the value of int_ActualHV should also follow.

The specification for verifying P1 is written as two  , reproduced below, which were verified in FDR. Here,  is a version of  where the unrelated events ext_pow24VStatus and int_pwmSignal are hidden, to ensure that the verification with respect to  and  is meaningful. It is verified  that ensures safety [14, p. 36], that is, an implementation    if, and only if, every behaviour of  is also a behaviour of  . Both of the assertions passed, thus successfully verifying property P1.

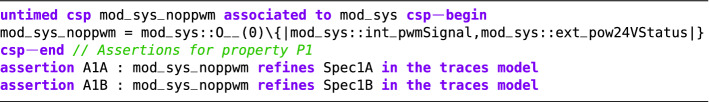



The second property (P2) requires that *PWM_Output* should be set to 0 when the 24 V power signal switches off. The CSP specification is shown below.

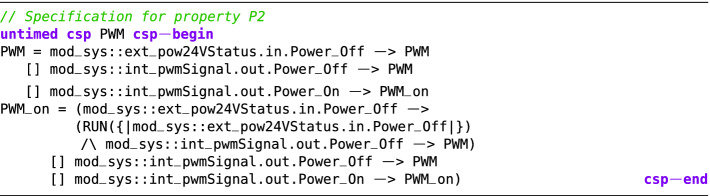



In  , there is an external choice  where the power status can be switched off via ext_pow24VStatus, or the int_pwmSignal may be switched Off. Once the int_pwmSignal becomes On, then the process behaves as  , where there is another choice: if the power status changes to Off via ext_pow24VStatus, then the output int_pwmSignal should follow as Off, while allowing further readings of the power status, specified using  , via ext_pow24VStatus, to take place. Observe that *P2* is specified by tracking the changes of int_pwmSignal relative to the input value of ext_pow24VStatus.

The assertion to verify the specification in FDR, as seen below, also passed, thus successfully verifying property P2, where  is a constrained form of  where other events are hidden similarly to previous assertions.

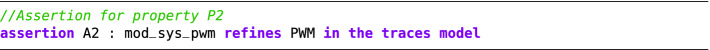



The third property to verify, P3, is that *HV_SetPoint* is set to 0 when the 24 power signal switches off. The specification in CSP is as follows.

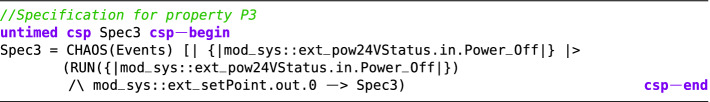



The assertion to verify P3 in FDR was written similarly to the ones for P1 and P2, as seen below. It passed in FDR, thus successfully verifying P3.

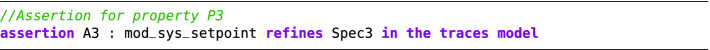



The assertion-specific language in RoboTool was used to specify and validate P4 and P5, which are that the state machine should be deadlock free and that all states should be reachable. The assertions written in RoboTool can be seen in the code below.

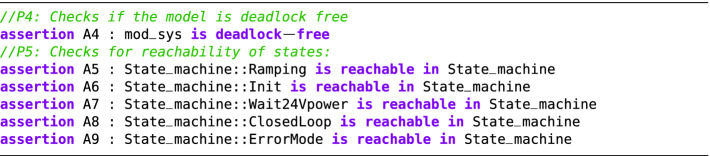



All these assertions passed in FDR, which implies that the state machine is in fact deadlock free, and all the states are reachable. Thus, also P4 and P5 were verified successfully.

### Model Checking in Simulink Design Verifier (SDV)

Simulink is a popular tool for traditional, input-driven simulation, and the modelling in SDV is similar to regular modelling used for simulation [5]. Thus, it requires less abstraction than in RoboTool, and it is more straightforward to translate the C++ code directly to the modelling language. The graphical language Stateflow, which is specifically created to model state machines, can be used with SDV, and was chosen to model the finite state machine from Sect. 3. SDV uses Prover Plug-In^®^ products from Prover^®^ Technology to do the model checking and prove the model properties [18]. It is built upon Gunnar Stålmarck’s proof procedure, which uses tautology checks to prove that an assertion holds true in every possible interpretation [19].

**Simplifications to Reduce Verification Time.** Also in the Simulink Design Verifier model, some simplifications had to be made in order to keep verification times reasonable. For example when modelling the transformer and CW generator from Fig. 2. The transformer is modelled as a simple transfer function, with non model-fitted values, poles and zeroes. The CW block is simply modelled with a gain block from the Simulink library, also this with an arbitrary value.

Additionally, the 24 V power signal and the PWM output signal have been simplified to being modelled binary, so they can be either on or off. Knowing if the signal is on or off is sufficient for verifying the properties listed for this use case.

**Fig. 10.**
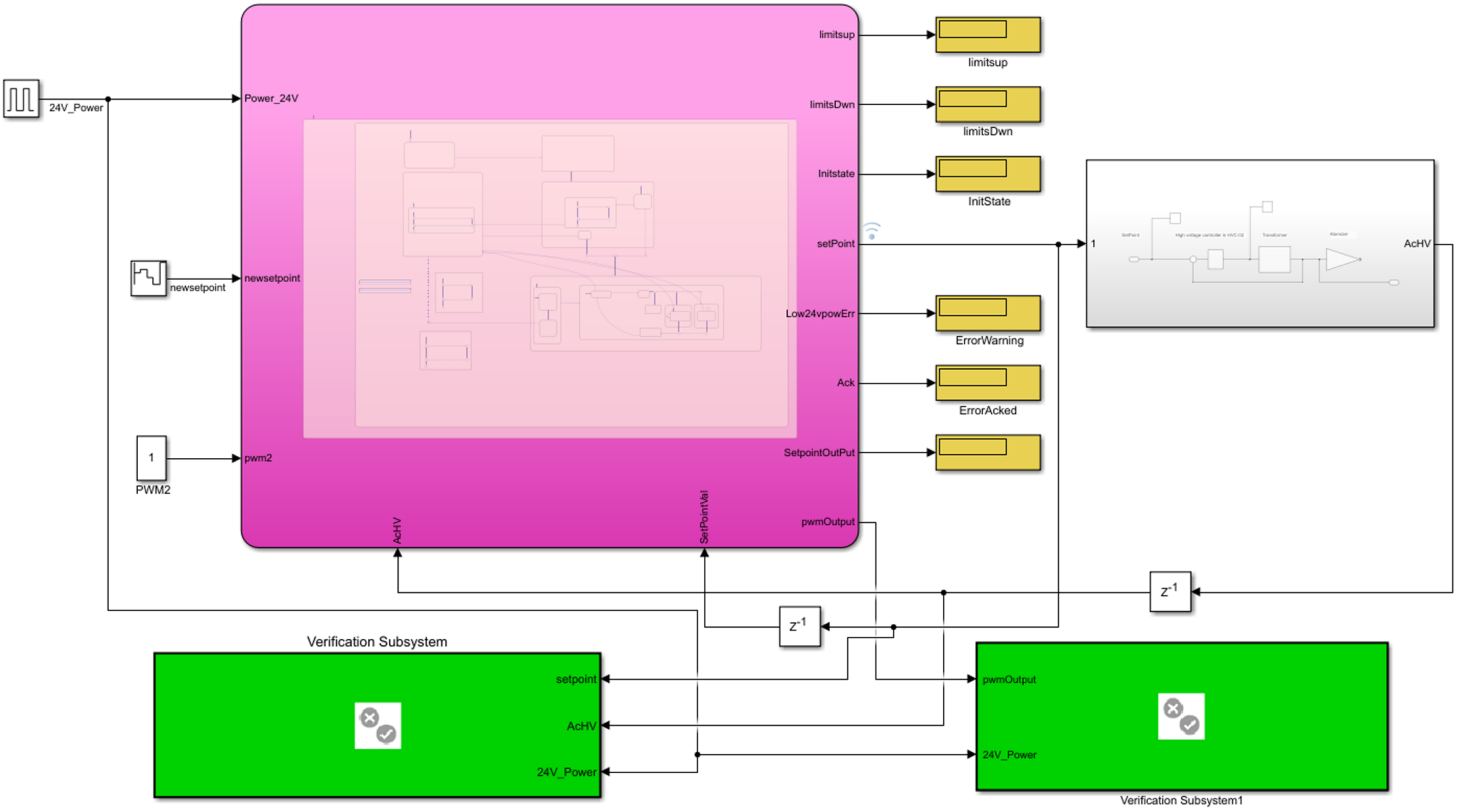
Overview of the Simulink model. (Color figure online)

**The Model.** An overview of the Simulink model can be seen in Fig. 10.

The main state machine from Fig. 4 can be found within the purple box, while variables, inputs and outputs are connected to it as seen in the figure. The grey box to the right contains the model of the transformer and CW block from Fig. 2. The green boxes are the verification subsystems containing the code for verifying the selected properties.

As seen in Fig. 10, the model used in SDV allows for defining inputs and outputs, similarly to a traditional simulation model. This made it possible to model the finite state machine very closely to the C++ code. However, some extra variables were introduced in order to model the ramping function.

**Fig. 11.**
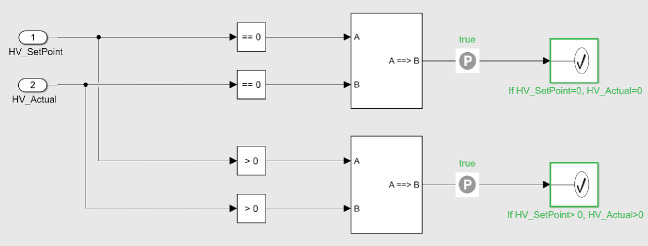
Property P1 modelled and verified in SDV. It is checked that whenever the value of *HV_SetPoint* is 0 or above 0, it implies the same for *HV_Actual*. The green rectangles to the right indicate that the properties were successfully verified. (Color figure online)

**Verification of Selected Properties.** In order to verify the properties from Sect. 2, the properties had to be formally modelled in SDV, using logical operator blocks. For verifying property P1, the following implications were considered and modelled in Simulink, as seen in Fig. 11:

$$\begin{aligned} \begin{aligned} HV\_SetPoint = 0&\rightarrow HV\_Actual = 0, \\ HV\_SetPoint> 0&\rightarrow HV\_Actual > 0. \end{aligned} \end{aligned}$$

For verifying property P2, the following implication was modelled in Simulink:

$$\begin{aligned} 24\,\mathrm{V}\_Power = 0 \rightarrow PWM\_Output = 0. \end{aligned}$$

**Fig. 12.**
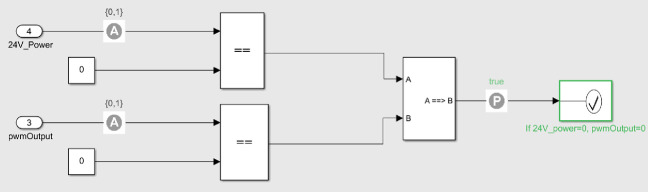
Property P2 modelled and verified in SDV. The assumption blocks assume the values of 24 V*_Power* and *PWM_Output* to be either 0 or 1, which is reasonable since they are modelled as binary. (Color figure online)

The assertion written in Simulink can be seen in Fig. 12. As indicated by the green rectangle to the right, also this property was successfully verified.

For verifying property P3, the following implication was modelled in Simulink:

$$\begin{aligned} \mathrm{24}\,\mathrm{V}\_Power = 0 \rightarrow HV\_SetPoint = 0. \end{aligned}$$

**Fig. 13.**
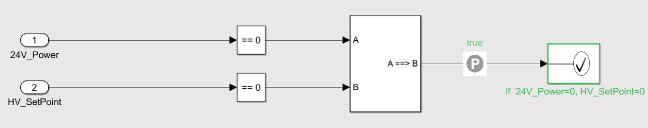
Property P3 modelled and verified in SDV.

The Simulink assertion can be seen in Fig. 13, and was successfully verified.

As far as SDV is considered, it does not offer the possibility to verify deadlock freedom^2^. Thus, P4 was not verified.

By using the Design Error Detection mode in SDV, it is possible to check the reachability of the states in the finite state machine and the results proved that all the states were reachable. Thus, P5 was verified successfully.

## Concluding Remarks and Future Work

As detailed in Sect. 4, some simplifications had to be made to the models used in RoboTool and SDV. In RoboTool, all properties were verified. Since SDV is not able to verify deadlock freedom, P4 was not verified in this tool.

The collective results formally show that all five specifications as listed in Sect. 2.1 are fulfilled, and thus the previously detected errors from Sect. 2 have been corrected. As these errors went undetected by traditional testing methods in an earlier version of the software, the results in this paper also serve as a testimony of the strength and suitability of using formal verification methods for industrial safety critical systems.

Both tool-chains offered the necessary functionality to model the HVC state machine and perform model checking. The verification times in both tools were typically about 2–3 min when running on a Windows laptop with Intel^®^ Core^©^ i5 CPU @ 2.71 GHz. These computation times are well within acceptable limits for offline, one-time verification purposes. However, it can be a potential bottleneck if used for development and debugging purposes.

As most industrial practitioners are used to using MathWorks products but are not as familiar with CSP, the use of SDV will most likely be the fastest and easiest way to do a formal verification. Unfamiliarity with CSP and RoboTool, which required a different way of thought and modelling, resulted in some challenges during both the modelling and verification processes. However, RoboTool has the advantage of being designed specifically for robotic systems, which gives it more targeted modelling options. During this work, we often learned about new functionalities and new ways to solve problems in RoboTool, which indicates that it is the better choice for more advanced use cases and experienced users.

To tap into this, future work involves further improvement of the RoboTool model. Instead of the simplifications made to the RoboTool model in order to reduce computation time, as mentioned in Sect. 4.1, it would be preferable to utilize distributed computation and parallel refinement checking capabilities of FDR. In this way, it could be possible to explore the possibility to include timed assertions and formulate requirements capturing the dynamic convergence of the *HV_Actual* to *HV_SetPoint*. By increasing the model’s complexity, future work will also look into how the tools perform with increasingly computationally heavy models. Also, the potential of using RoboSim to generate (verified) simulations from RoboChart, is currently being further investigated.

As far as MathWorks is considered, the Polyspace software tools provide some complementary verification capabilities, such as deadlock detection, that should be examined and utilized in future work. In order to continue the comparative study, updates and improvements will be made to the Simulink model as well in future work, to ensure a fair comparison with RoboTool. This includes, for instance, formulating a specification property that captures the dynamics of the set-point following.

In this work, formal verification has been used on the already rectified code, in order to ensure that the errors have been corrected. However, to further demonstrate the abilities of formal verification as a supplement to testing, it would be interesting to repeat the process on the faulty code. This would increase the confidence that our modelling captures the behaviour properly, by showing that the errors would have been detected earlier if formal verification had been applied. Thus, future work will include repeating the verification steps on the faulty code as well.

## Footnotes

1 https://github.com/robo-star/hvc-case-study.

2 Deadlock detection is instead provided by MathWorks Polyspace toolbox.
